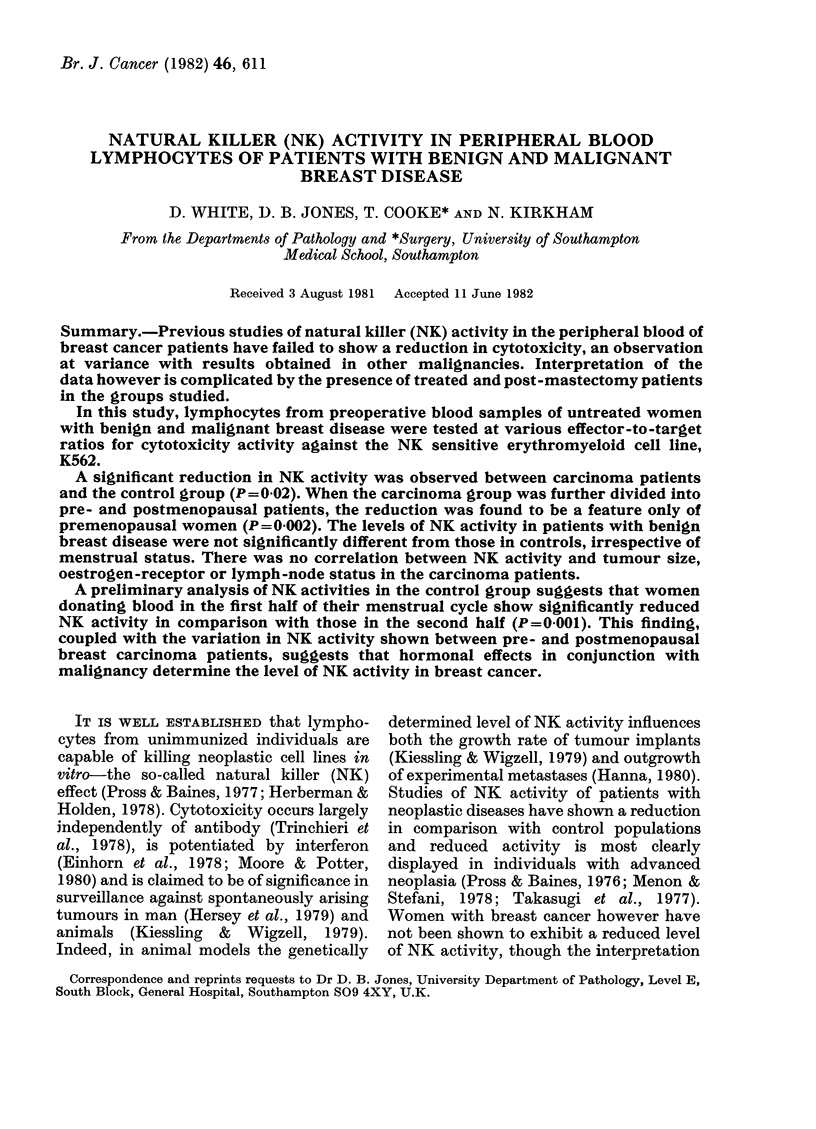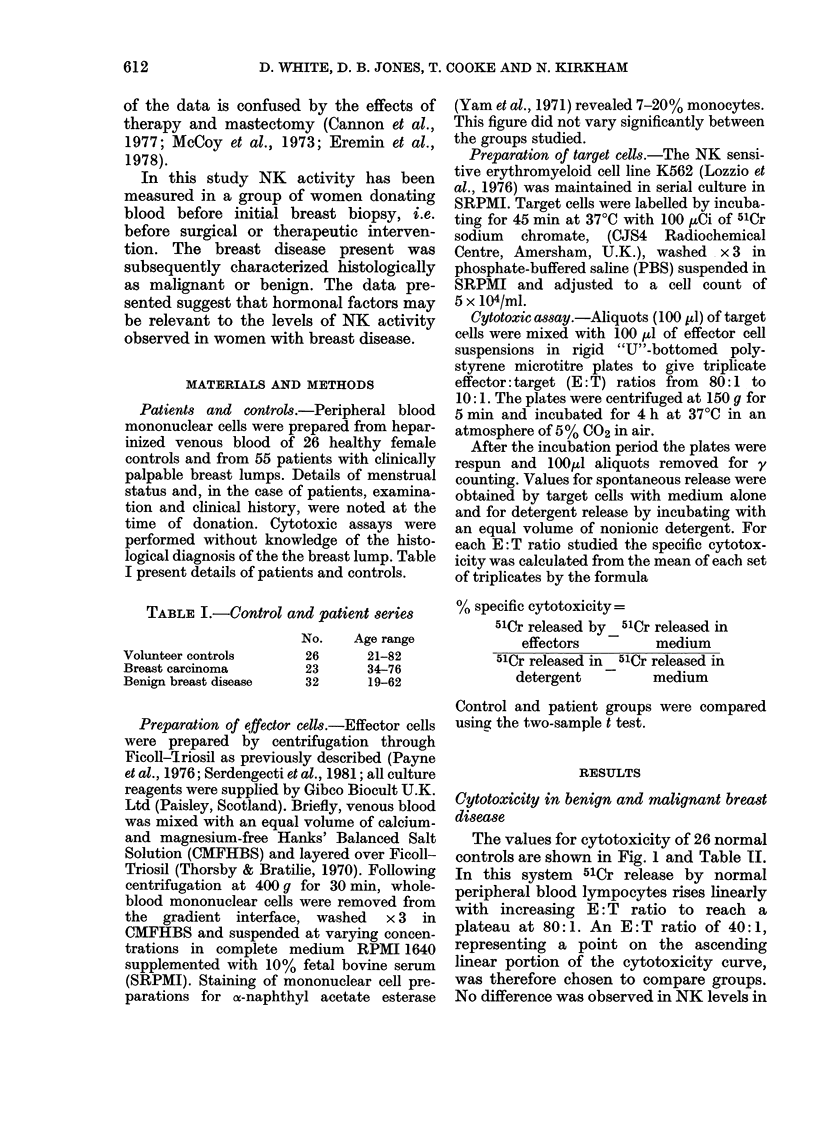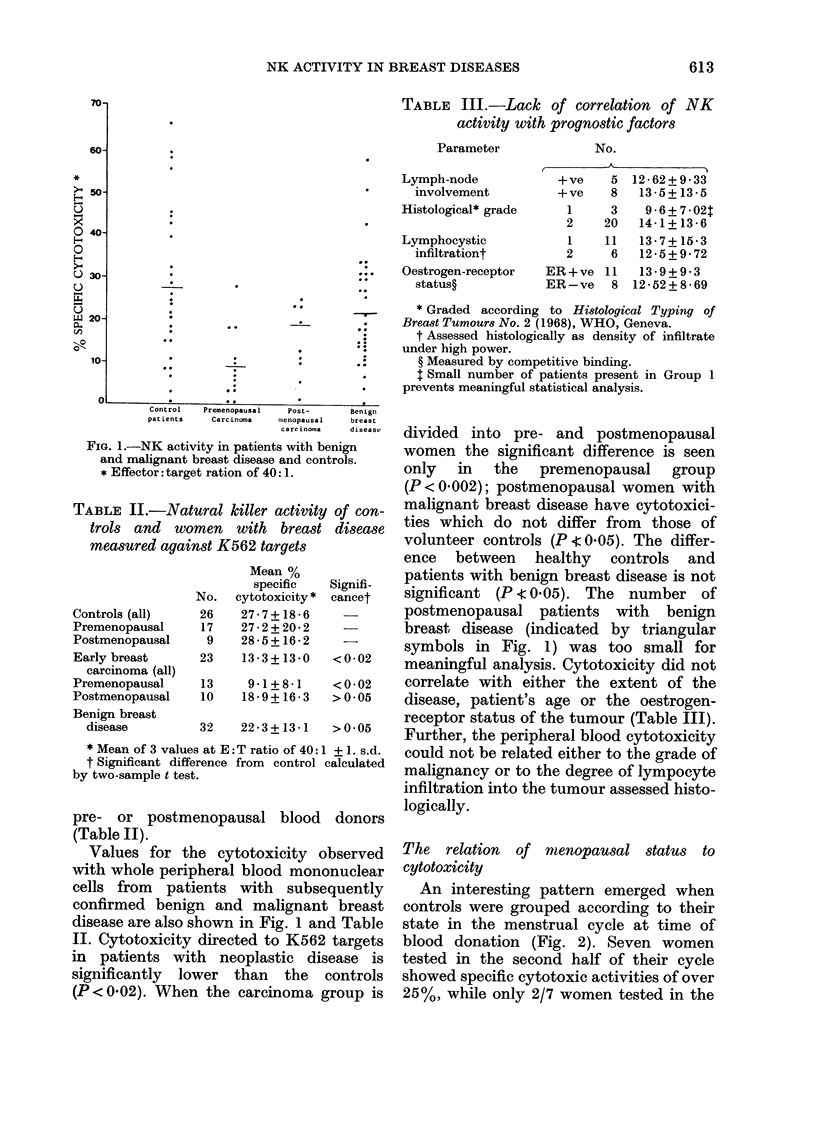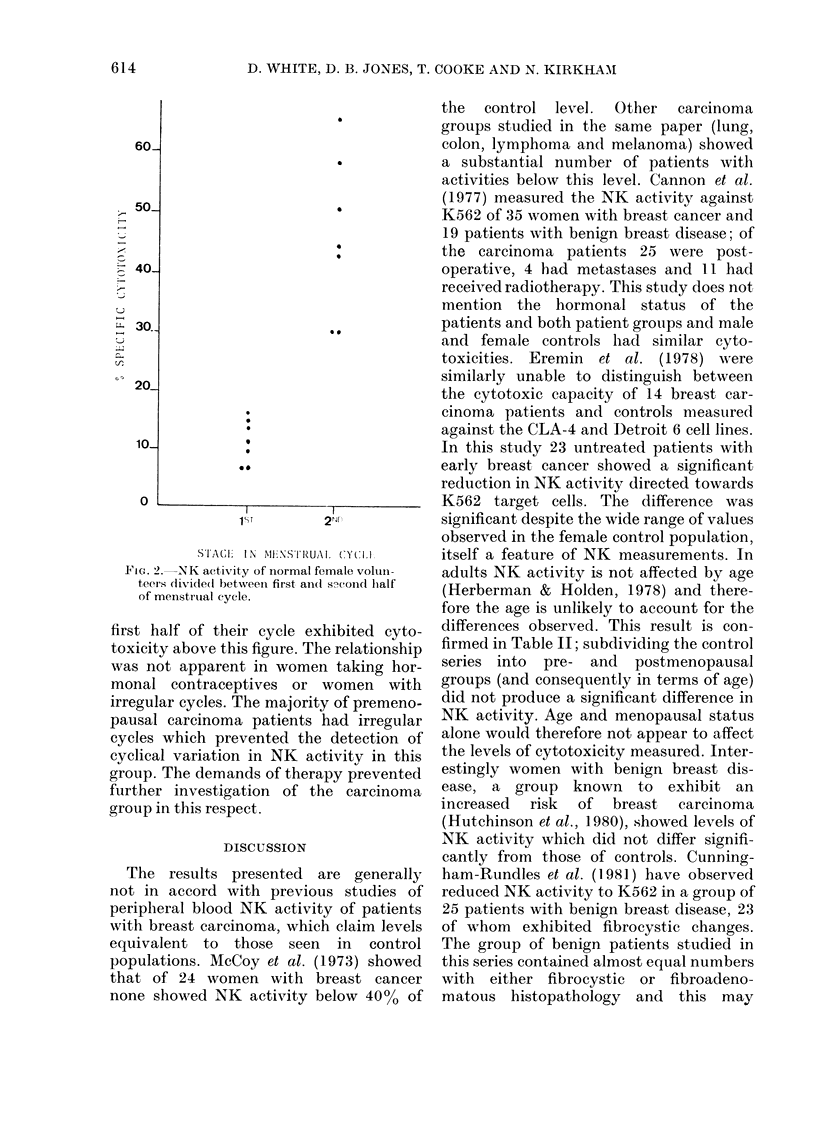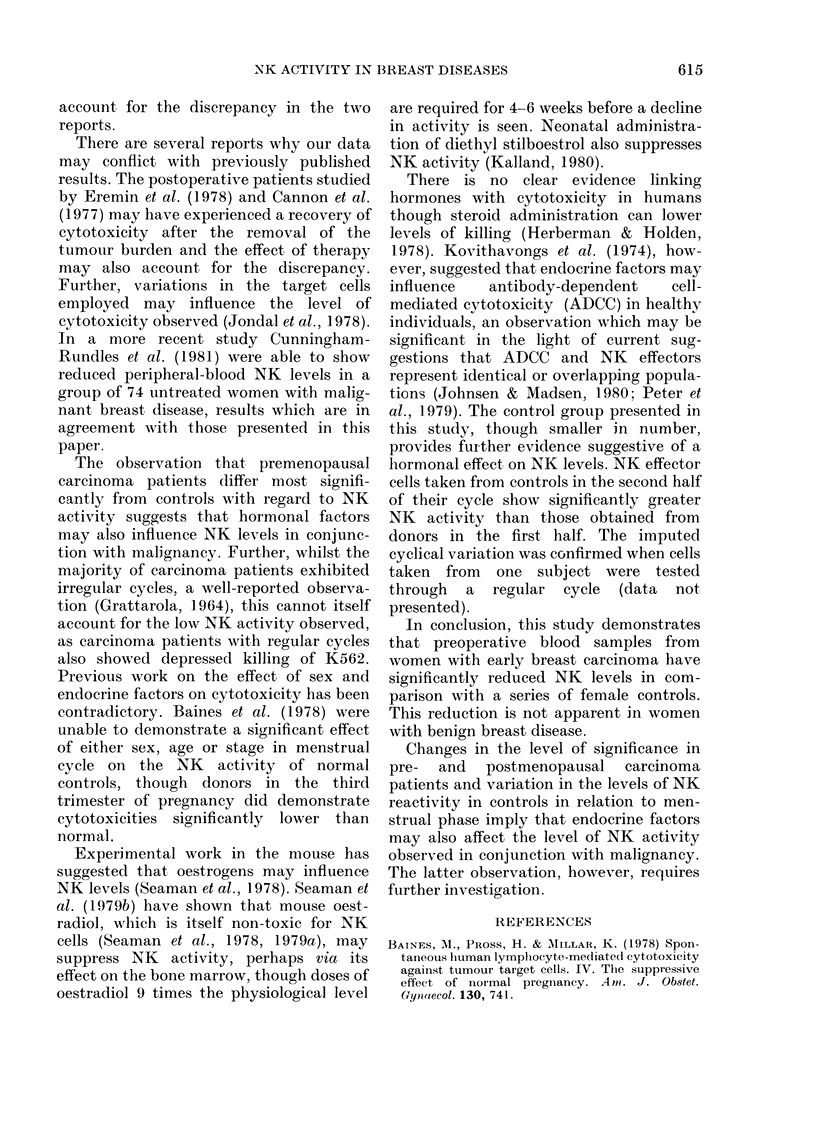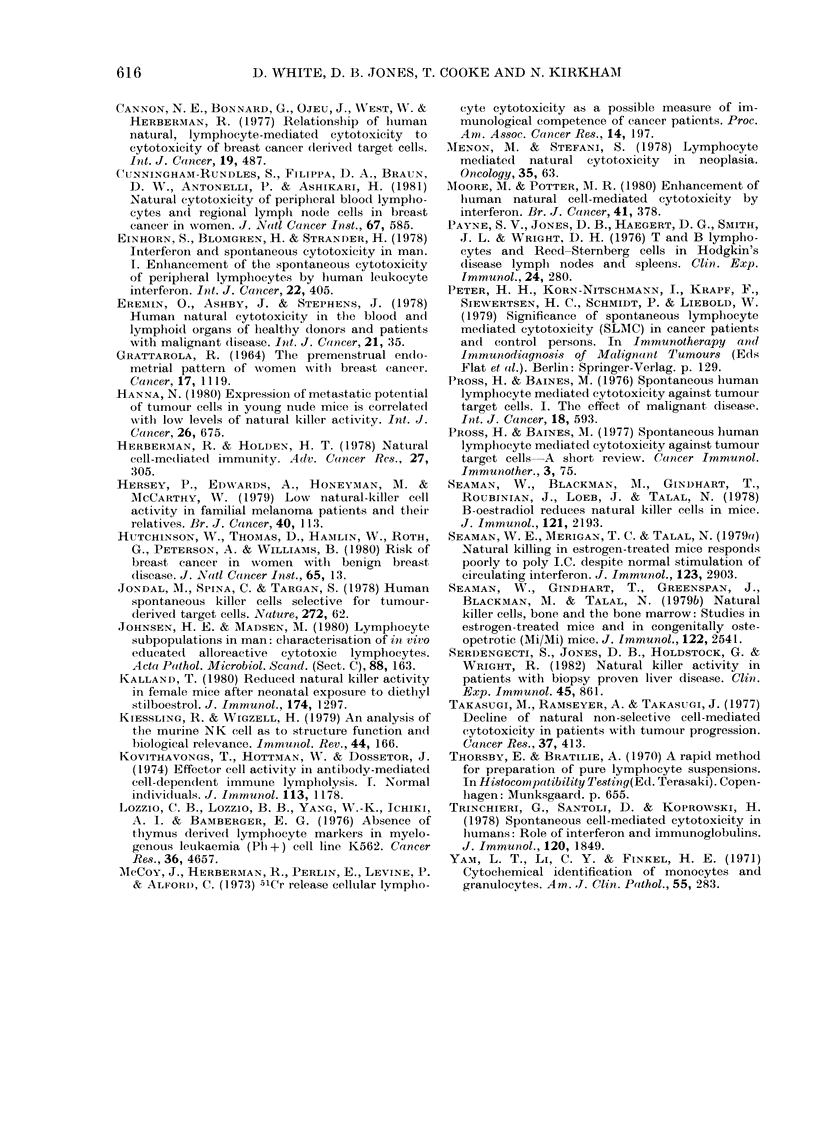# Natural killer (NK) activity in peripheral blood lymphocytes of patients with benign and malignant breast disease

**DOI:** 10.1038/bjc.1982.245

**Published:** 1982-10

**Authors:** D. White, D. B. Jones, T. Cooke, N. Kirkham

## Abstract

Previous studies of natural killer (NK) activity in the peripheral blood of breast cancer patients have failed to show a reduction in cytotoxicity, an observation at variance with results obtained in other malignancies. Interpretation of the data however is complicated by the presence of treated and post-mastectomy patients in the groups studied.

In this study, lymphocytes from preoperative blood samples of untreated women with benign and malignant breast disease were tested at various effector-to-target ratios for cytotoxicity activity against the NK sensitive erythromyeloid cell line, K562.

A significant reduction in NK activity was observed between carcinoma patients and the control group (*P*=0·02). When the carcinoma group was further divided into pre- and postmenopausal patients, the reduction was found to be a feature only of premenopausal women (*P*=0·002). The levels of NK activity in patients with benign breast disease were not significantly different from those in controls, irrespective of menstrual status. There was no correlation between NK activity and tumour size, oestrogen-receptor or lymph-node status in the carcinoma patients.

A preliminary analysis of NK activities in the control group suggests that women donating blood in the first half of their menstrual cycle show significantly reduced NK activity in comparison with those in the second half (*P*=0·001). This finding, coupled with the variation in NK activity shown between pre- and postmenopausal breast carcinoma patients, suggests that hormonal effects in conjunction with malignancy determine the level of NK activity in breast cancer.


*Br. J. Cancer* (1982) **46**, 611

### NATURAL KILLER (NK) ACTIVITY IN PERIPHERAL BLOOD LYMPHOCYTES OF PATIENTS WITH BENIGN AND MALIGNANT BREAST DISEASE

D. WHITE, D. B. JONES, T. COOKE* AND N. KIRKHAM

*From the Departments of Pathology and *Surgery, University of Southampton Medical School, Southampton*

Received 3 August 1981 Accepted 11 June 1982

**Summary.—Previous studies of natural killer (NK) activity in the peripheral blood of breast cancer patients have failed to show a reduction in cytotoxicity, an observation at variance with results obtained in other malignancies. Interpretation of the data however is complicated by the presence of treated and post-mastectomy patients in the groups studied.**

**In this study, lymphocytes from preoperative blood samples of untreated women with benign and malignant breast disease were tested at various effector-to-target ratios for cytotoxicity activity against the NK sensitive erythromyeloid cell line, K562.**

**A significant reduction in NK activity was observed between carcinoma patients and the control group ($P=0.02$). When the carcinoma group was further divided into pre- and postmenopausal patients, the reduction was found to be a feature only of premenopausal women ($P=0.002$). The levels of NK activity in patients with benign breast disease were not significantly different from those in controls, irrespective of menstrual status. There was no correlation between NK activity and tumour size, oestrogen-receptor or lymph-node status in the carcinoma patients.**

**A preliminary analysis of NK activities in the control group suggests that women donating blood in the first half of their menstrual cycle show significantly reduced NK activity in comparison with those in the second half ($P=0.001$). This finding, coupled with the variation in NK activity shown between pre- and postmenopausal breast carcinoma patients, suggests that hormonal effects in conjunction with malignancy determine the level of NK activity in breast cancer.**

IT IS WELL ESTABLISHED that lymphocytes from unimmunized individuals are capable of killing neoplastic cell lines *in vitro*—the so-called natural killer (NK) effect (Pross & Baines, 1977; Herberman & Holden, 1978). Cytotoxicity occurs largely independently of antibody (Trinchieri *et al.*, 1978), is potentiated by interferon (Einhorn *et al.*, 1978; Moore & Potter, 1980) and is claimed to be of significance in surveillance against spontaneously arising tumours in man (Hersey *et al.*, 1979) and animals (Kiessling & Wigzell, 1979). Indeed, in animal models the genetically determined level of NK activity influences both the growth rate of tumour implants (Kiessling & Wigzell, 1979) and outgrowth of experimental metastases (Hanna, 1980). Studies of NK activity of patients with neoplastic diseases have shown a reduction in comparison with control populations and reduced activity is most clearly displayed in individuals with advanced neoplasia (Pross & Baines, 1976; Menon & Stefani, 1978; Takasugi *et al.*, 1977). Women with breast cancer however have not been shown to exhibit a reduced level of NK activity, though the interpretation

Correspondence and reprints requests to Dr D. B. Jones, University Department of Pathology, Level E, South Block, General Hospital, Southampton SO9 4XY, U.K.

of the data is confused by the effects of therapy and mastectomy (Cannon *et al.*, 1977; McCoy *et al.*, 1973; Eremin *et al.*, 1978).

In this study NK activity has been measured in a group of women donating blood before initial breast biopsy, *i.e.* before surgical or therapeutic intervention. The breast disease present was subsequently characterized histologically as malignant or benign. The data presented suggest that hormonal factors may be relevant to the levels of NK activity observed in women with breast disease.

### MATERIALS AND METHODS

*Patients and controls.*—Peripheral blood mononuclear cells were prepared from heparinized venous blood of 26 healthy female controls and from 55 patients with clinically palpable breast lumps. Details of menstrual status and, in the case of patients, examination and clinical history, were noted at the time of donation. Cytotoxic assays were performed without knowledge of the histological diagnosis of the the breast lump. Table I present details of patients and controls.

TABLE I.—*Control and patient series*

| | No. | Age range |
|---|---|---|
| Volunteer controls | 26 | 21–82 |
| Breast carcinoma | 23 | 34–76 |
| Benign breast disease | 32 | 19–62 |

*Preparation of effector cells.*—Effector cells were prepared by centrifugation through Ficoll–Triosil as previously described (Payne *et al.*, 1976; Serdengecti *et al.*, 1981; all culture reagents were supplied by Gibco Biocult U.K. Ltd (Paisley, Scotland). Briefly, venous blood was mixed with an equal volume of calcium- and magnesium-free Hanks' Balanced Salt Solution (CMFHBS) and layered over Ficoll–Triosil (Thorsby & Bratilie, 1970). Following centrifugation at 400 *g* for 30 min, whole-blood mononuclear cells were removed from the gradient interface, washed ×3 in CMFHBS and suspended at varying concentrations in complete medium RPMI 1640 supplemented with 10% fetal bovine serum (SRPMI). Staining of mononuclear cell preparations for α-naphthyl acetate esterase (Yam *et al.*, 1971) revealed 7–20% monocytes. This figure did not vary significantly between the groups studied.

*Preparation of target cells.*—The NK sensitive erythromyeloid cell line K562 (Lozzio *et al.*, 1976) was maintained in serial culture in SRPMI. Target cells were labelled by incubating for 45 min at 37°C with 100 μCi of $^{51}Cr$ sodium chromate, (CJS4 Radiochemical Centre, Amersham, U.K.), washed ×3 in phosphate-buffered saline (PBS) suspended in SRPMI and adjusted to a cell count of $5 \times 10^4$/ml.

*Cytotoxic assay.*—Aliquots (100 μl) of target cells were mixed with 100 μl of effector cell suspensions in rigid "U"-bottomed polystyrene microtitre plates to give triplicate effector:target (E:T) ratios from 80:1 to 10:1. The plates were centrifuged at 150 *g* for 5 min and incubated for 4 h at 37°C in an atmosphere of 5% $CO_2$ in air.

After the incubation period the plates were respun and 100μl aliquots removed for γ counting. Values for spontaneous release were obtained by target cells with medium alone and for detergent release by incubating with an equal volume of nonionic detergent. For each E:T ratio studied the specific cytotoxicity was calculated from the mean of each set of triplicates by the formula

$$\% \text{ specific cytotoxicity} = \frac{{}^{51}\text{Cr released by effectors} - {}^{51}\text{Cr released in medium}}{{}^{51}\text{Cr released in detergent} - {}^{51}\text{Cr released in medium}}$$

Control and patient groups were compared using the two-sample *t* test.

### RESULTS

### *Cytotoxicity in benign and malignant breast disease*

The values for cytotoxicity of 26 normal controls are shown in Fig. 1 and Table II. In this system $^{51}Cr$ release by normal peripheral blood lympocytes rises linearly with increasing E:T ratio to reach a plateau at 80:1. An E:T ratio of 40:1, representing a point on the ascending linear portion of the cytotoxicity curve, was therefore chosen to compare groups. No difference was observed in NK levels in

FIG. 1.—NK activity in patients with benign and malignant breast disease and controls. * Effector: target ration of 40:1.

TABLE II.—*Natural killer activity of controls and women with breast disease measured against K562 targets*

| | No. | Mean % specific cytotoxicity* | Significance† |
|---|---|---|---|
| Controls (all) | 26 | 27·7±18·6 | — |
| Premenopausal | 17 | 27·2±20·2 | — |
| Postmenopausal | 9 | 28·5±16·2 | — |
| Early breast carcinoma (all) | 23 | 13·3±13·0 | <0·02 |
| Premenopausal | 13 | 9·1±8·1 | <0·02 |
| Postmenopausal | 10 | 18·9±16·3 | >0·05 |
| Benign breast disease | 32 | 22·3±13·1 | >0·05 |

* Mean of 3 values at E:T ratio of 40:1 ±1. s.d.

† Significant difference from control calculated by two-sample *t* test.

pre- or postmenopausal blood donors (Table II).

Values for the cytotoxicity observed with whole peripheral blood mononuclear cells from patients with subsequently confirmed benign and malignant breast disease are also shown in Fig. 1 and Table II. Cytotoxicity directed to K562 targets in patients with neoplastic disease is significantly lower than the controls ($P < 0·02$). When the carcinoma group is

TABLE III.—*Lack of correlation of NK activity with prognostic factors*

| Parameter | | No. | |
|---|---|---|---|
| Lymph-node involvement | +ve | 5 | 12·62±9·33 |
| | +ve | 8 | 13·5±13·5 |
| Histological* grade | 1 | 3 | 9·6±7·02‡ |
| | 2 | 20 | 14·1±13·6 |
| Lymphocystic infiltration† | 1 | 11 | 13·7±15·3 |
| | 2 | 6 | 12·5±9·72 |
| Oestrogen-receptor status§ | ER+ve | 11 | 13·9±9·3 |
| | ER−ve | 8 | 12·52±8·69 |

* Graded according to *Histological Typing of Breast Tumours No.* 2 (1968), WHO, Geneva.
† Assessed histologically as density of infiltrate under high power.
§ Measured by competitive binding.
‡ Small number of patients present in Group 1 prevents meaningful statistical analysis.

divided into pre- and postmenopausal women the significant difference is seen only in the premenopausal group ($P < 0·002$); postmenopausal women with malignant breast disease have cytotoxicities which do not differ from those of volunteer controls ($P \nless 0·05$). The difference between healthy controls and patients with benign breast disease is not significant ($P \nless 0·05$). The number of postmenopausal patients with benign breast disease (indicated by triangular symbols in Fig. 1) was too small for meaningful analysis. Cytotoxicity did not correlate with either the extent of the disease, patient's age or the oestrogen-receptor status of the tumour (Table III). Further, the peripheral blood cytotoxicity could not be related either to the grade of malignancy or to the degree of lympocyte infiltration into the tumour assessed histologically.

*The relation of menopausal status to cytotoxicity*

An interesting pattern emerged when controls were grouped according to their state in the menstrual cycle at time of blood donation (Fig. 2). Seven women tested in the second half of their cycle showed specific cytotoxic activities of over 25%, while only 2/7 women tested in the

FIG. 2.—NK activity of normal female volunteers divided between first and second half of menstrual cycle.

first half of their cycle exhibited cytotoxicity above this figure. The relationship was not apparent in women taking hormonal contraceptives or women with irregular cycles. The majority of premenopausal carcinoma patients had irregular cycles which prevented the detection of cyclical variation in NK activity in this group. The demands of therapy prevented further investigation of the carcinoma group in this respect.

### DISCUSSION

The results presented are generally not in accord with previous studies of peripheral blood NK activity of patients with breast carcinoma, which claim levels equivalent to those seen in control populations. McCoy *et al.* (1973) showed that of 24 women with breast cancer none showed NK activity below 40% of the control level. Other carcinoma groups studied in the same paper (lung, colon, lymphoma and melanoma) showed a substantial number of patients with activities below this level. Cannon *et al.* (1977) measured the NK activity against K562 of 35 women with breast cancer and 19 patients with benign breast disease; of the carcinoma patients 25 were post-operative, 4 had metastases and 11 had received radiotherapy. This study does not mention the hormonal status of the patients and both patient groups and male and female controls had similar cytotoxicities. Eremin *et al.* (1978) were similarly unable to distinguish between the cytotoxic capacity of 14 breast carcinoma patients and controls measured against the CLA-4 and Detroit 6 cell lines. In this study 23 untreated patients with early breast cancer showed a significant reduction in NK activity directed towards K562 target cells. The difference was significant despite the wide range of values observed in the female control population, itself a feature of NK measurements. In adults NK activity is not affected by age (Herberman & Holden, 1978) and therefore the age is unlikely to account for the differences observed. This result is confirmed in Table II; subdividing the control series into pre- and postmenopausal groups (and consequently in terms of age) did not produce a significant difference in NK activity. Age and menopausal status alone would therefore not appear to affect the levels of cytotoxicity measured. Interestingly women with benign breast disease, a group known to exhibit an increased risk of breast carcinoma (Hutchinson *et al.*, 1980), showed levels of NK activity which did not differ significantly from those of controls. Cunningham-Rundles *et al.* (1981) have observed reduced NK activity to K562 in a group of 25 patients with benign breast disease, 23 of whom exhibited fibrocystic changes. The group of benign patients studied in this series contained almost equal numbers with either fibrocystic or fibroadenomatous histopathology and this may account for the discrepancy in the two reports.

There are several reports why our data may conflict with previously published results. The postoperative patients studied by Eremin *et al.* (1978) and Cannon *et al.* (1977) may have experienced a recovery of cytotoxicity after the removal of the tumour burden and the effect of therapy may also account for the discrepancy. Further, variations in the target cells employed may influence the level of cytotoxicity observed (Jondal *et al.*, 1978). In a more recent study Cunningham-Rundles *et al.* (1981) were able to show reduced peripheral-blood NK levels in a group of 74 untreated women with malignant breast disease, results which are in agreement with those presented in this paper.

The observation that premenopausal carcinoma patients differ most significantly from controls with regard to NK activity suggests that hormonal factors may also influence NK levels in conjunction with malignancy. Further, whilst the majority of carcinoma patients exhibited irregular cycles, a well-reported observation (Grattarola, 1964), this cannot itself account for the low NK activity observed, as carcinoma patients with regular cycles also showed depressed killing of K562. Previous work on the effect of sex and endocrine factors on cytotoxicity has been contradictory. Baines *et al.* (1978) were unable to demonstrate a significant effect of either sex, age or stage in menstrual cycle on the NK activity of normal controls, though donors in the third trimester of pregnancy did demonstrate cytotoxicities significantly lower than normal.

Experimental work in the mouse has suggested that oestrogens may influence NK levels (Seaman *et al.*, 1978). Seaman *et al.* (1979*b*) have shown that mouse oestradiol, which is itself non-toxic for NK cells (Seaman *et al.*, 1978, 1979*a*), may suppress NK activity, perhaps *via* its effect on the bone marrow, though doses of oestradiol 9 times the physiological level are required for 4–6 weeks before a decline in activity is seen. Neonatal administration of diethyl stilboestrol also suppresses NK activity (Kalland, 1980).

There is no clear evidence linking hormones with cytotoxicity in humans though steroid administration can lower levels of killing (Herberman & Holden, 1978). Kovithavongs *et al.* (1974), however, suggested that endocrine factors may influence antibody-dependent cell-mediated cytotoxicity (ADCC) in healthy individuals, an observation which may be significant in the light of current suggestions that ADCC and NK effectors represent identical or overlapping populations (Johnsen & Madsen, 1980; Peter *et al.*, 1979). The control group presented in this study, though smaller in number, provides further evidence suggestive of a hormonal effect on NK levels. NK effector cells taken from controls in the second half of their cycle show significantly greater NK activity than those obtained from donors in the first half. The imputed cyclical variation was confirmed when cells taken from one subject were tested through a regular cycle (data not presented).

In conclusion, this study demonstrates that preoperative blood samples from women with early breast carcinoma have significantly reduced NK levels in comparison with a series of female controls. This reduction is not apparent in women with benign breast disease.

Changes in the level of significance in pre- and postmenopausal carcinoma patients and variation in the levels of NK reactivity in controls in relation to menstrual phase imply that endocrine factors may also affect the level of NK activity observed in conjunction with malignancy. The latter observation, however, requires further investigation.

### REFERENCES

BAINES, M., PROSS, H. & MILLAR, K. (1978) Spontaneous human lymphocyte-mediated cytotoxicity against tumour target cells. IV. The suppressive effect of normal pregnancy. *Am. J. Obstet. Gynaecol.* **130,** 741.

CANNON, N. E., BONNARD, G., OJEU, J., WEST, W. & HERBERMAN, R. (1977) Relationship of human natural, lymphocyte-mediated cytotoxicity to cytotoxicity of breast cancer derived target cells. *Int. J. Cancer*, **19**, 487.

CUNNINGHAM-RUNDLES, S., FILIPPA, D. A., BRAUN, D. W., ANTONELLI, P. & ASHIKARI, H. (1981) Natural cytotoxicity of peripheral blood lymphocytes and regional lymph node cells in breast cancer in women. *J. Natl Cancer Inst.*, **67**, 585.

EINHORN, S., BLOMGREN, H. & STRANDER, H. (1978) Interferon and spontaneous cytotoxicity in man. I. Enhancement of the spontaneous cytotoxicity of peripheral lymphocytes by human leukocyte interferon. *Int. J. Cancer*, **22**, 405.

EREMIN, O., ASHBY, J. & STEPHENS, J. (1978) Human natural cytotoxicity in the blood and lymphoid organs of healthy donors and patients with malignant disease. *Int. J. Cancer*, **21**, 35.

GRATTAROLA, R. (1964) The premenstrual endometrial pattern of women with breast cancer. *Cancer*, **17**, 1119.

HANNA, N. (1980) Expression of metastatic potential of tumour cells in young nude mice is correlated with low levels of natural killer activity. *Int. J. Cancer*, **26**, 675.

HERBERMAN, R. & HOLDEN, H. T. (1978) Natural cell-mediated immunity. *Adv. Cancer Res.*, **27**, 305.

HERSEY, P., EDWARDS, A., HONEYMAN, M. & McCARTHY, W. (1979) Low natural-killer cell activity in familial melanoma patients and their relatives. *Br. J. Cancer*, **40**, 113.

HUTCHINSON, W., THOMAS, D., HAMLIN, W., ROTH, G., PETERSON, A. & WILLIAMS, B. (1980) Risk of breast cancer in women with benign breast disease. *J. Natl Cancer Inst.*, **65**, 13.

JONDAL, M., SPINA, C. & TARGAN, S. (1978) Human spontaneous killer cells selective for tumour-derived target cells. *Nature*, **272**, 62.

JOHNSEN, H. E. & MADSEN, M. (1980) Lymphocyte subpopulations in man: characterisation of *in vivo* educated alloreactive cytotoxic lymphocytes. *Acta Pathol. Microbiol. Scand.* (Sect. C), **88**, 163.

KALLAND, T. (1980) Reduced natural killer activity in female mice after neonatal exposure to diethyl stilboestrol. *J. Immunol.*, **174**, 1297.

KIESSLING, R. & WIGZELL, H. (1979) An analysis of the murine NK cell as to structure function and biological relevance. *Immunol. Rev.*, **44**, 166.

KOVITHAVONGS, T., HOTTMAN, W. & DOSSETOR, J. (1974) Effector cell activity in antibody-mediated cell-dependent immune lympholysis. I. Normal individuals. *J. Immunol.* **113**, 1178.

LOZZIO, C. B., LOZZIO, B. B., YANG, W.-K., ICHIKI, A. I. & BAMBERGER, E. G. (1976) Absence of thymus derived lymphocyte markers in myelogenous leukaemia (Ph+) cell line K562. *Cancer Res.*, **36**, 4657.

McCOY, J., HERBERMAN, R., PERLIN, E., LEVINE, P. & ALFORD, C. (1973) $^{51}$Cr release cellular lymphocyte cytotoxicity as a possible measure of immunological competence of cancer patients. *Proc. Am. Assoc. Cancer Res.*, **14**, 197.

MENON, M. & STEFANI, S. (1978) Lymphocyte mediated natural cytotoxicity in neoplasia. *Oncology*, **35**, 63.

MOORE, M. & POTTER, M. R. (1980) Enhancement of human natural cell-mediated cytotoxicity by interferon. *Br. J. Cancer*, **41**, 378.

PAYNE, S. V., JONES, D. B., HAEGERT, D. G., SMITH, J. L. & WRIGHT, D. H. (1976) T and B lymphocytes and Reed–Sternberg cells in Hodgkin's disease lymph nodes and spleens. *Clin. Exp. Immunol.*, **24**, 280.

PETER, H. H., KORN-NITSCHMANN, I., KRAPF, F., SIEWERTSEN, H. C., SCHMIDT, P. & LIEBOLD, W. (1979) Significance of spontaneous lymphocyte mediated cytotoxicity (SLMC) in cancer patients and control persons. In *Immunotherapy and Immunodiagnosis of Malignant Tumours* (Eds Flat *et al.*). Berlin: Springer-Verlag. p. 129.

PROSS, H. & BAINES, M. (1976) Spontaneous human lymphocyte mediated cytotoxicity against tumour target cells. I. The effect of malignant disease. *Int. J. Cancer*, **18**, 593.

PROSS, H. & BAINES, M. (1977) Spontaneous human lymphocyte mediated cytotoxicity against tumour target cells—A short review. *Cancer Immunol. Immunother.*, **3**, 75.

SEAMAN, W., BLACKMAN, M., GINDHART, T., ROUBINIAN, J., LOEB, J. & TALAL, N. (1978) B-oestradiol reduces natural killer cells in mice. *J. Immunol.*, **121**, 2193.

SEAMAN, W. E., MERIGAN, T. C. & TALAL, N. (1979*a*) Natural killing in estrogen-treated mice responds poorly to poly I.C. despite normal stimulation of circulating interferon. *J. Immunol.*, **123**, 2903.

SEAMAN, W., GINDHART, T., GREENSPAN, J., BLACKMAN, M. & TALAL, N. (1979*b*) Natural killer cells, bone and the bone marrow: Studies in estrogen-treated mice and in congenitally osteopetrotic (Mi/Mi) mice. *J. Immunol.*, **122**, 2541.

SERDENGECTI, S., JONES, D. B., HOLDSTOCK, G. & WRIGHT, R. (1982) Natural killer activity in patients with biopsy proven liver disease. *Clin. Exp. Immunol.* **45**, 861.

TAKASUGI, M., RAMSEYER, A. & TAKASUGI, J. (1977) Decline of natural non-selective cell-mediated cytotoxicity in patients with tumour progression. *Cancer Res.*, **37**, 413.

THORSBY, E. & BRATILIE, A. (1970) A rapid method for preparation of pure lymphocyte suspensions. In *Histocompatibility Testing* (Ed. Terasaki). Copenhagen: Munksgaard. p. 655.

TRINCHIERI, G., SANTOLI, D. & KOPROWSKI, H. (1978) Spontaneous cell-mediated cytotoxicity in humans: Role of interferon and immunoglobulins. *J. Immunol.*, **120**, 1849.

YAM, L. T., LI, C. Y. & FINKEL, H. E. (1971) Cytochemical identification of monocytes and granulocytes. *Am. J. Clin. Pathol.*, **55**, 283.